# Impact of p53-associated acute myeloid leukemia hallmarks on metabolism and the immune environment

**DOI:** 10.3389/fphar.2024.1409210

**Published:** 2024-08-05

**Authors:** Monika Chomczyk, Luca Gazzola, Shubhankar Dash, Patryk Firmanty, Binsah S. George, Vakul Mohanty, Hussein A. Abbas, Natalia Baran

**Affiliations:** ^1^ Department of Experimental Hematology, Institute of Hematology and Transfusion Medicine, Warsaw, Poland; ^2^ Department of Biomedical and Neuromotor Sciences, University of Bologna, Bologna, Italy; ^3^ Department of Hematology-oncology, The University of Texas Health Sciences, Houston, TX, United States; ^4^ Department of Bioinformatics and Computational Biology, The University of Texas MD Anderson Cancer Center, Houston, TX, United States; ^5^ Department of Leukemia, The University of Texas MD Anderson Cancer Center, Houston, TX, United States

**Keywords:** AML, *TP53* mutations, drug resistance, immunosuppression, metabolic rewiring, therapeutic approaches

## Abstract

Acute myeloid leukemia **(**AML), an aggressive malignancy of hematopoietic stem cells, is characterized by the blockade of cell differentiation, uncontrolled proliferation, and cell expansion that impairs healthy hematopoiesis and results in pancytopenia and susceptibility to infections. Several genetic and chromosomal aberrations play a role in AML and influence patient outcomes. *TP53* is a key tumor suppressor gene involved in a variety of cell features, such as cell-cycle regulation, genome stability, proliferation, differentiation, stem-cell homeostasis, apoptosis, metabolism, senescence, and the repair of DNA damage in response to cellular stress. In AML, *TP53* alterations occur in 5%–12% of *de novo* AML cases. These mutations form an important molecular subgroup, and patients with these mutations have the worst prognosis and shortest overall survival among patients with AML, even when treated with aggressive chemotherapy and allogeneic stem cell transplant. The frequency of *TP53-*mutations increases in relapsed and recurrent AML and is associated with chemoresistance. Progress in AML genetics and biology has brought the novel therapies, however, the clinical benefit of these agents for patients whose disease is driven by *TP53* mutations remains largely unexplored. This review focuses on the molecular characteristics of *TP53*-mutated disease; the impact of *TP53* on selected hallmarks of leukemia, particularly metabolic rewiring and immune evasion, the clinical importance of *TP53* mutations; and the current progress in the development of preclinical and clinical therapeutic strategies to treat *TP53*-mutated disease.

## 1 Introduction

### 1.1 Acute myeloid leukemia

Acute myeloid leukemia (AML) is a malignant, clonal, hematological disease (Caruso et al., 2022) that develops from transformed hematopoietic stem cells (HSCs) and is characterized by cells’ uncontrolled proliferation, expansion, and an unlimited capacity for self-renewal (George et al., 2021). AML occurs mainly in adults over 40 years old, with the peak incidence in patients above 70 years of age (Daver et al., 2023a). Most patients with AML harbor mutations that cause cells’ malignant proliferation and enhance their ability to evade cell death (Chen et al., 2022). AML shows different metabolic and physiologic hallmarks, depending on the types of harbored mutations (Kennedy and Lowe, 2022). The most commonly mutated genes related to AML initiation and progression include FMS-related receptor tyrosine kinase 3 (*FLT3*), DNA methyltransferase three alpha (*DNMT3A*), nucleophosmin 1 (*NPM1*), and tet methylcytosine dioxygenase 2 (*TET2*) (Villatoro et al., 2020). AML mutations, together with cytogenetic abnormalities, have critical implications for clinical outcomes (Olivier et al., 2010). It is estimated that 50% of *de novo* AML cases show cytogenetic abnormalities, and the number and frequency of mutations increase in patients developing therapy-related AML, who have been treated previously with cytotoxic therapies such as alkylating agents, topoisomerase II inhibitors, and radiotherapy (Hong et al., 2016). Therapy-related AML is also frequently characterized by complex karyotypes and mutations in the tumor protein p53 (*TP53*) gene (Olivier et al., 2010; Hong et al., 2016). The incidence of *TP53* mutations in AML can vary. While the frequency of *TP53* mutations is estimated to account for 10% of *de novo* AML cases, it rises strikingly in therapy-related AML or relapsing/refractory (R/R) AML cases, reaching up to 30% and 25%, respectively, in these groups (Daver et al., 2023a). An even higher frequency of mutant *TP53* is associated with the complex karyotype subtype of AML, in which the frequency of *TP53* mutations reaches up to 70%, mainly due to selective pressure caused by acquired resistance to DNA damage following chemotherapy and radiotherapy.

Identifying potential therapeutic vulnerabilities and therapeutic targets in AML is challenging due to AML’s genetic heterogeneity (Zhou et al., 2020). The exploration of novel genomic targets has led to the development of only a few potent targeted therapies, such as IDH, FLT3, or KMT2A inhibitors for genomically defined AML subsets (Appelbaum et al., 2024). However, due to the genotypic and phenotypic diversity of mutant *TP53,* finding targeted therapies against *TP53* remains an unresolved challenge.

### 1.2 TP53

*TP53* is a 20-kbp gene located on chromosome 17p13.1 (Olivier et al., 2010). So far, 15 isoforms of p53 have been identified (Haaland et al., 2021). Despite some differences, each p53 isoform consists of five common domains: an *N*-terminal, a proline-rich domain, a DNA-binding domain (DBD), a regulatory domain, and a C-terminal (George et al., 2021). Activation of p53 occurs in response to diverse cellular stress factors such as hypoxia, DNA damage, oncogene expression, or replicative stress (Daver et al., 2022; Wang H. et al., 2023). Through its DBD domain, p53 regulates the transcription of genes involved in cell-cycle regulation, genome stability, proliferation, stem-cell homeostasis and differentiation, and cell-death regulation (Daver et al., 2022; Wang H. et al., 2023). P53 also has antiangiogenic properties; it represses metastases, controls tumor-promoting inflammation, facilitates the immune response, promotes replicative senescence, enhances the effects of growth suppressors, and regulates energetics and metabolism (Daver et al., 2022; Wang H. et al., 2023). The p53 inhibitors MDM2 and MDM4, which control p53 s ubiquitination and ubiquitin–proteasome system activity, tightly regulate p53 levels. (Wang H. et al., 2023). In AML, p53 is mainly silenced by the upregulation of MDM2, MDM4/MDMX, ARF, and E6 (Abramowitz et al., 2017; Latif et al., 2021; Tashakori et al., 2022). The precisely controlled level of p53 might be also disturbed due to somatic mutations in *TP53* or to imbalances in the gene products interacting with p53, leading to its inactivation (Olivier et al., 2010). Most somatic mutations occur mainly as point missense mutations, frameshift insertions or deletions, splice sites, and nonsense mutations; these mutations are observed in leukemia and in many other types of cancer (Olivier et al., 2010). Studies have shown that almost 90% of *TP53* mutations detected in patients with therapy-related myeloid neoplasms have variant allele frequencies (VAFs) greater than 10%, and that these VAFs frequently occurred with the loss of 17p across the *TP53* locus (loss of heterozygosity) or as copy-neutral loss of heterozygosity (Donehower et al., 2019; Shah et al., 2023). Compared to wild-type *TP53*, *TP53* mutation with a VAF greater than10% was associated with inferior outcomes and worse survival (Donehower et al., 2019; Shah et al., 2023).

*TP53* mutations are a strong indicator of prognosis, and studies have shown that, in AML, multi-hit mutated *TP53* is associated with genomic instability, thrombocytopenia, and a higher blast count, independent of the VAF (Deng et al., 2020). Further studies have shown that, unlike AML cells that carry multi-hit mutated *TP53*, those that carry monoallelic *TP53* mutations frequently harbor co-mutations in genes like tet methylcytosine dioxygenase 2 (*TET2*), splicing factor 3b subunit 1 (*SF3B1*), ASXL transcriptional regulator 1 (*ASXL1*), RUNX family transcription factor 1 (*RUNX1*), isocitrate dehydrogenase (NADP(+)) 2 (*IDH2*), serine and arginine rich splicing factor 2 (*SRSF2)* (Shah et al., 2023), Mitogen-activated protein kinase kinase kinase 7 (*TAK1*), BCL6 corepressor (*BCOR*), and Cbl proto-oncogene (*CBL*) (Daver et al., 2023a). Finally, *TP53* monoallelic mutations with co-mutated *RUNX1*, KRAS proto-oncogene, GTPase (*KRAS*), or *CBL* are correlated with poor prognosis more frequently than monoallelic *TP53* mutations (Daver et al., 2023a). Although performing a *TP53* status analysis is not yet considered standard procedure, Cox multivariate hazard models have shown that heavy alterations of *TP53* allele status independently predict a poor prognosis (Daver et al., 2023a). The 2022 findings of European LeukemiaNet support the consideration of a *TP53* mutation as a distinct AML entity with a “very-adverse” risk profile like that listed for European LeukemiaNet in the 2022 International Consensus Classification (Tashakori et al., 2022; Fleming et al., 2023).

#### 1.2.1 Alteration of the *TP53* gene in AML and other cancers

In AML, *TP53* mutations are mainly found in 6 DBD hotspots: R175H, G245S, R248Q/W, R249S, R273H/S, and R282W (Kennedy and Lowe, 2022). These mutations lead to reduced activity, complete loss of function (LOF), or, less frequently, to a switch or gain of function (GOF), suggesting that there may be some tissue-specific requirements for the loss of wild-type (wt) or gain of mutant p53 functions (Boettcher et al., 2019; Daver et al., 2022; Kennedy and Lowe, 2022).

Beyond the most common *TP53* mutations, the Y220C mutation frequently appears in various solid tumors and leukemias (Barnoud et al., 2021). Research suggests that this mutation is the ninth-most-frequent p53 cancer mutation (Barnoud et al., 2021; Hassin and Oren, 2023). It creates a cavity on the p53’s surface, making it highly unstable. Interestingly, the Y220C mutation has been linked to 3 cases of familial cancer, and it appears to grant the p53 new cancer-promoting abilities. These abilities include stimulating the growth of blood vessels (angiogenesis) and making cancer cells resistant to the chemotherapy drug doxorubicin (Loke et al., 2022).

**FIGURE 1:**
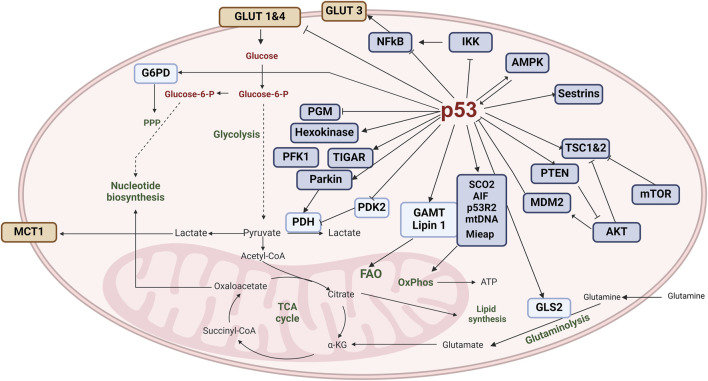
The role of p53 in the hallmarks of acute myeloid leukemia. The wild-type and mutant forms of p53 play distinct roles in controlling the diverse hallmarks of cancer. Wild-type p53 (wtp53) maintains effective anti-tumor immunity, mainly through its role in cell death (apoptosis or autophagy), senescence, or regulation of reactive oxygen species (ROS). It is also involved in regulating the immune response. In contrast, mutant p53 exerts negative effects on the immune environment by activating inflammatory pathways; this process can be enhanced by an excessive production of ROS. In consequence, mutant p53 contributes to cancer-associated chronic inflammation and protects leukemia cells from immune-mediated destruction and phagocytosis. It causes genome instability and the accumulation of new mutations that further perturbate the DNA repair machinery and enable uncontrolled growth and arrest of the cell cycle. Mutant p53 also sustains proliferative signaling, leading to replicative immortality, resistance to cell death, and finally metabolic rewiring to meet new energetic and biosynthetic demands. (Image created with BioRender.com **)**.

In general, *TP53* mutations substantially impact tumor development. In fact, over half of all human cancers have some form of *TP53* mutation. The mutations can be particularly severe in Li-Fraumeni syndrome, in which a mutated *TP53* gene dramatically increases the risk of cancers like osteosarcoma, leukemia, breast cancer, brain tumors, and adrenal tumors (Vaddavalli and Schumacher, 2022). Since p53 plays a role in many cellular processes, mutations in this gene can disrupt a wide range of functions and ultimately make cells more likely to acquire the characteristics needed to become cancerous, as illustrated in Figure 1.

Mutations in *TP53* affect homeostasis, not only via altering protein activity, but also by altering the isoforms ratio, which may diminish a patient’s response to chemotherapy (Haaland et al., 2021). Among the most common *TP53* mutations, mutations in the hotspots R175 and R248 are frequently detected in diverse solid tumors but occur less frequently in AML (Barnoud et al., 2021)*.* Interestingly, AML patients with heterozygous *TP53* mutations have shown similar responses to therapy as those harboring wt *TP53* (Barnoud et al., 2021; Kennedy and Lowe, 2022; Daver et al., 2023a). However, the sequential acquisition of the mutation of one allele, followed by mutation of the other allele or loss of the entire 17p chromosome, leads to a complete LOF of wt *TP53* and has been identified as the key progression mechanism involving *TP53* mutation. (Hong et al., 2016; Boettcher et al., 2019; Yang et al., 2022; Shah et al., 2023). Furthermore, wt *TP53* LOF is seen in patients with Li-Fraumeni syndrome; affected individuals develop AML at a frequency comparable to that of healthy individuals with other *TP53* mutations, but they manifest more aggressive disease (Prokocimer et al., 2017; Boettcher et al., 2019; Daver et al., 2022; Shah et al., 2023). *TP53* LOF is often correlated with nonautonomous effects on the tumor immune microenvironment; it subverts the wt p53 effect and allows the evasion of attack from the immune system (Prokocimer et al., 2017; Loizou et al., 2019; Hassin and Oren, 2023; Rajagopalan et al., 2023). Many point mutations in *TP53* have been studied by overexpressing the missense allele in *TP53* null tumor cells; specifically, an increase in growth independence, tumor progression, metastasis, and drug resistance (Singh et al., 2019). These changes have been associated with missense *TP53* variants, indicating that they have novel functions that promote tumor growth and contribute to tumorigenesis. (Loizou et al., 2019; Singh et al., 2019; Rajagopalan et al., 2023). While the GOF of mutated p53 in AML harboring *TP53* mut/mut is still debated, an alternative mechanism called separation of function seems to contribute to AML pathogenesis (Kennedy and Lowe, 2022). Table 1 summarizes the two main types of mutations that can affect the p53 protein, a critical tumor suppressor in the human body. Understanding these mutations leads to a fuller understanding of how cancers develop and progress.

**TABLE 1:** Differences between the loss-of-function and gain-of-function mutations affecting the p53 protein.

	Mutations affecting the p53 protein	
Mutation characteristic	LOF	GOF
**Frequency**	Common	Less frequent
**Effect**	Mutations inactivate the p53 protein and decreased its ability to suppress tumor growth	Mutations inactivate p53’s tumor-suppressor function and promote tumor growth
**Mechanism**	Mutations occur in various ways (e.g., insertions/deletions of genetic material, point mutations that change amino acids in the protein). These changes disrupt the protein’s structure or its ability to bind to DNA, hindering its function as a tumor suppressor	Mutations often occur in the DNA-binding domain of p53. These mutations alter how the protein interacts with DNA, allowing it to bind to different genetic sites and regulate genes that favor tumorigenesis
**Impact**	Cells with nonfunctional p53 protein lack the normal cell-cycle arrest or apoptosis (programmed cell death) mechanisms triggered by DNA damage	GOF-mutant p53 can promote metastasis, induce resistance to therapy, and help cancer cells evade the immune system
**Summary**	LOF mutations prevent cells from damaged DNA	GOF mutations alter DNA binding, which not only makes cells ineffective at their original functions but also allows them to actively assist cancer progression

GOF, gain-of-function; LOF, loss-of-function.

#### 1.2.2 p53 phosphorylation status and its role in AML

p53 activity is tightly regulated by posttranslational modifications, with phosphorylation being a crucial event (Ashcroft et al., 1999). Understanding p53 phosphorylation at multiple serine, threonine, and tyrosine residues by specific kinases [ataxia telangiectasia mutated (ATM), ataxia telangiectasia and Rad3-related (ATR), and checkpoint kinase 2 (CHK2)] in response to diverse cellular stresses (e.g., DNA damage, oxidative stress) and examining the multifaceted consequences of p53 phosphorylation, including enhanced protein stability, augmented transcriptional activity, and modulation of subcellular localization, may lead to the development of new therapeutic strategies (Sakaguchi et al., 1998; Ashcroft et al., 1999). These phosphorylation events orchestrate the activation of various target genes involved in cell-cycle arrest, DNA repair, and apoptosis, ultimately determining cellular fate following stress induction (Loughery and Meek, 2013). A significant percentage of AML cases have mutations in the *TP53* gene, and these mutations can disrupt the normal phosphorylation and regulation of p53, leading to its dysfunction (Ni et al., 2024). Mutant p53 proteins may have altered phosphorylation patterns or impaired interactions with kinases and other regulatory proteins, affecting their ability to properly respond to cellular stresses (Hales et al., 2014).

Several signaling pathways, such as the PI3K/AKT/mTOR pathway, MAPK pathway, and JAK/STAT pathway, can modulate p53 phosphorylation and activity in leukemias (Leu et al., 2020). Dysregulation of these pathways in AML can influence p53 phosphorylation and its downstream functions, potentially promoting leukemic cell survival or drug resistance (Motlagh et al., 2022).

## 2 The role of p53 in the hallmarks of leukemia

p53 is a master regulator of cancer-relevant pathways, governing genomic stability (DNA repair), cell fate (cell cycle arrest, senescence, apoptosis), and cellular processes (metabolism, autophagy, ferroptosis) (Figure 1) (Singh et al., 2019).

### 2.1 *TP53* and the regulation of energetics, metabolism, and metabolic reprogramming

**FIGURE 2:**
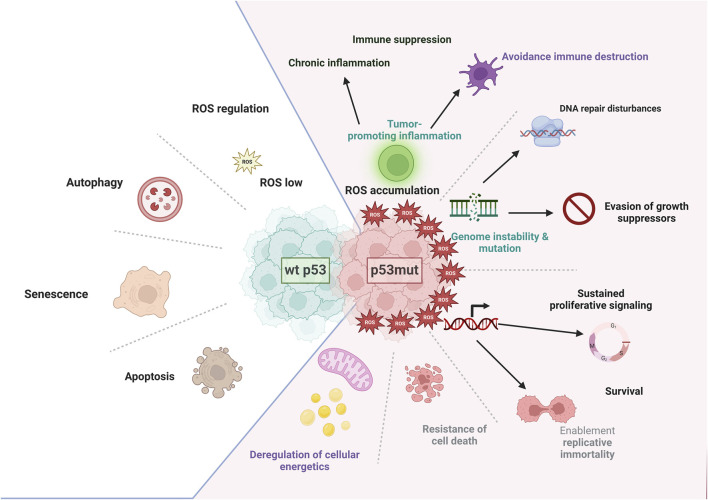
p53 and metabolism. A crucial protein in cellular regulation, p53 exerts control over several metabolic pathways involved in energy production and cellular homeostasis. It also plays a substantial role in regulating mitochondrial oxidative phosphorylation, glycolysis, glutaminolysis, fatty-acid oxidation, and nucleotide synthesis. To maintain mitochondrial integrity and promote oxidative phosphorylation, p53 transcriptionally induces the expression of SCO2, AIF1, and p53R2, while also physically interacting with mitochondrial DNA polymerase γ and MIAP (mitochondrial import associated protein). Additionally, p53 reduces glucose uptake by directly repressing the transcription of GLUT1 and GLUT4 and indirectly repressing the expression of GLUT3. Furthermore, p53 negatively regulates phosphoglycerate mutase, activates hexokinase and phosphofructokinase one at the protein level, and transcriptionally induces *TP53*-induced glycolysis and apoptosis regulator (TIGAR) and Parkin, which inhibit glycolysis. Parkin, in turn, positively regulates pyruvate dehydrogenase and the expression of monocarboxylate transporter 1. In contrast, p53 negatively regulates the expression of pyruvate dehydrogenase kinase isoform 2, which inhibits PDH activity. It also induces the expression of glutaminase 2 (GLS2), which catalyzes the hydrolysis of glutamine to glutamate. Glutamate is converted to α-ketoglutarate, which promotes the tricarboxylic acid (TCA) cycle and oxidative phosphorylation. p53 also interacts with glucose-6-phosphate dehydrogenase (G6PD) to negatively regulate its activity, leading to the downregulation of the pentose phosphate pathway, nucleotide synthesis, and nicotinamide adenine dinucleotide phosphate (NADPH) production. p53 induces the expression of GAMT and Lipin1, promoting fatty-acid oxidation and the production of acetyl-CoA. (Image created with BioRender.com).

The *TP53* gene plays a crucial role in regulating cellular energy and metabolism (Leu et al., 2020). As a transcription factor, *TP53* controls the expression of various genes involved in metabolic pathways (Motlagh et al., 2022; Luo et al., 2023). It influences the balance between glycolysis and oxidative phosphorylation (Motlagh et al., 2022; Luo et al., 2023). *TP53* also impacts glycolysis through reduction or downregulation of key glycolytic enzymes, or transporters for glucose, pyruvate or other essential for glycolysis nutrients, and through suppression of the AKT/mTOR and NF-κB signaling pathways. (Motlagh et al., 2022; Luo et al., 2023; McClure et al., 2023; Roche et al., 2023). Additionally, *TP53* controls glucose-regulating cellular energetics and metabolism by suppressing the glucose transporters (GLUT1 and GLUT4) that bring glucose into the cell. *TP53* can also induce *TP53*-induced glycolysis and apoptosis regulator, which diverts glucose away from glycolysis and towards the pentose-phosphate pathway, involved in nucleotide synthesis, lipids synthesis, and amino acids synthesis to meet the energy demands of the cell (Motlagh et al., 2022; Luo et al., 2023; McClure et al., 2023; Roche et al., 2023). *TP53* can induce the expression of genes involved in antioxidant defense, protecting cells from oxidative stress (Sakaguchi et al., 1998; Ashcroft et al., 1999; Loughery and Meek, 2013; Hales et al., 2014; Ni et al., 2024). This shift towards oxidative phosphorylation increases ATP production and provides more efficient energy for cellular processes (Luo et al., 2023; McClure et al., 2023; Roche et al., 2023). *TP53* also inhibits the expression of genes involved in fatty-acid synthesis, promoting lipid breakdown and utilization (Luo et al., 2023; McClure et al., 2023; Roche et al., 2023). Furthermore, *TP53* can modulate the activity of enzymes involved in energy metabolism, such as AMP-activated protein kinase (AMPK) (Motlagh et al., 2022; Luo et al., 2023; McClure et al., 2023; Roche et al., 2023). In addition, it can influence mitochondrial function and biogenesis, affecting cellular energy production (Motlagh et al., 2022; Luo et al., 2023; McClure et al., 2023; Roche et al., 2023; Sanford et al., 2023; Li et al., 2024). Overall, *TP53* plays a multifaceted role in cellular energy and metabolism, maintaining the balance between energy production and utilization and the protection against cellular stress. Dysregulation of *TP53* can lead to metabolic reprogramming, which is often observed in cancer and other diseases (Leu et al., 2020; Motlagh et al., 2022; Luo et al., 2023; Roche et al., 2023) and can also lead to decreased mitochondrial activity, further compromising energy production and forcing cells to increasingly rely on glycolysis, which is inefficient (Figure 2) (Motlagh et al., 2022; Luo et al., 2023; Roche et al., 2023).

### 2.2 *TP53,* the immune response, immune evasion, immunosuppression, and tumor-promoting inflammation

*TP53* mutations can lead to the downregulation of major histocompatibility complex (MHC) molecules, which present antigens to immune cells (Alos et al., 2020; Motlagh et al., 2022; Hassin and Oren, 2023). By reducing MHC expression, cancer cells can evade recognition by cytotoxic T cells, which rely on MHC-antigen complexes to identify and eliminate abnormal cells (Alos et al., 2020; Wang et al., 2024). Compared to patients with TP53-WT, patients who have AML with *TP53* mutations have shown higher expression levels of *IFNG, FOXP3* in blast cells of primary BM samples, immune checkpoints, markers of immune senescence, and phosphatidylinositol 3-kinase-Akt and NF-κB signaling intermediates (Vadakekolathu et al., 2020). *TP53* mutations can also impair the activation of T cells, which are crucial for mounting an effective immune response against cancer cells (Desai et al., 2023; Wang et al., 2024). *TP53* regulates the expression of costimulatory molecules and cytokines involved in T-cell activation (Desai et al., 2023; Wang et al., 2024). Mutations in *TP53* can disrupt this regulation, leading to insufficient T-cell activation and compromised antitumor immune responses (Alos et al., 2020; Desai et al., 2023). *TP53* mutations can also negatively impact the effector functions of immune cells such as cytotoxic T cells and natural killer (NK) cells (Alos et al., 2020). These mutations can result in the downregulation of cytotoxic molecules such as perforin and granzyme B, which are responsible for killing cancer cells (Desai et al., 2023). As a result, cancer cells can evade immune-mediated cell death.

Programmed cell death protein one and its ligand, programmed cell death ligand 1 (PD-L1), play a role in immune tolerance and the suppression of antitumor immune responses, and *TP53* has been shown to regulate the expression of these immune checkpoint molecules (Alos et al., 2020). Dysregulation of *TP53* can lead to abnormal expression of PD-L1, which can inhibit T-cell function and promote immune evasion (Alos et al., 2020). *TP53* mutations induce immunosuppressive factors such as transforming growth factor beta and interleukin-10 (Daver et al., 2021). These factors can inhibit the activation and function of immune cells, creating an immunosuppressive microenvironment that favors tumor growth and immune evasion (Alos et al., 2020; Daver et al., 2021). *TP53* mutations also can disrupt the normal processes of tumor immune surveillance, which is the mechanism by which the immune system detects and eliminates cancer cells (Alos et al., 2020; Daver et al., 2021).

*TP53* can activate immune cells such as macrophages, dendritic cells, and NK cells (Motlagh et al., 2022). It regulates the expression of cytokines such as interferons and interleukins, chemokines, and costimulatory molecules involved in immune-cell activation, immune signaling, immune cell function, and coordination (Motlagh et al., 2022). Proper T-cell activation is crucial for an effective immune response (Hassin and Oren, 2023; Wang et al., 2024). *TP53* controls the recruitment of immune cells to the site of infection or inflammation by regulating the expression of chemokines (Wang et al., 2024). This helps in mobilizing immune cells and directing them to the specific locations where they are needed (Wang et al., 2024). In addition, *TP53* influences the differentiation of immune cells such as macrophages and dendritic cells, which are responsible for phagocytosis, antigen presentation, and immune regulation, and ensures an effective immune response (Singh et al., 2019; Motlagh et al., 2022; Wang et al., 2024).

**FIGURE 3:**
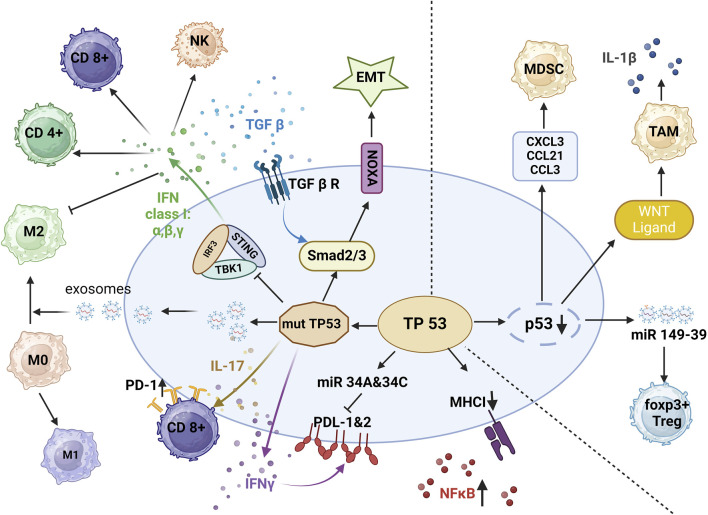
p53 and immune suppression. In the setting of mutant p53, decreased release of IFN-I reduces infiltration by CD4^+^ T cells, CD8^+^ T cells, and natural killer (NK) cells, and interferon gamma (IFNγ) increases the expression of programmed cell death ligand 1 (PD-L1) and programmed cell death ligand 2 (PD-L2). By activation of interleukin 17 (IL-17) signaling, mutant p53 leads to CD8^+^T-cell exhaustion. In concert with transforming growth factor beta (TGF-β), mutant p53 promotes epithelial-to-mesenchymal transition EMT. With the release of exosomes, mutant p53 also promotes the polarization of M1 macrophages to M2 macrophages, protecting tumor cells from phagocytosis. In the setting of p53 deletion, tumor cells release WNT ligands, chemokine ligand 1 (CXCL1), chemokine ligand 3 (CCL3) and chemokine ligand 21 (CCL21), and miR-149–39 to enhance the differentiation of tumor-associated macrophages (TAMs) and regulatory T cells (Tregs) (Image created with BioRender.com).

Tp53 participates in tumor immune surveillance and is involved in the resolution of inflammation by promoting the clearance of inflammatory cells and the restoration of tissue homeostasis. This helps in preventing chronic inflammation, which can have detrimental effects on the immune system (Alos et al., 2020). Tumor-promoting inflammation, also known as chronic inflammation, is caused by an overproduction of proinflammatory cytokines such as tumor necrosis factor-α, interleukin-1, and interleukin-6 (Shi and Jiang, 2021; Morganti et al., 2022; Miller et al., 2023; Muto et al., 2023; Qin et al., 2023). These cytokines can promote tumor growth by stimulating cell proliferation, angiogenesis, and tissue remodeling (Shi and Jiang, 2021; Morganti et al., 2022; Muto et al., 2023; Qin et al., 2023). Immune cells, including macrophages, neutrophils, and lymphocytes, release additional proinflammatory molecules, creating a positive feedback loop that sustains inflammation and promotes tumor growth (Herbrich et al., 2021; Muto et al., 2023; Qin et al., 2023). Inflammatory cells release reactive oxygen species and reactive nitrogen species, which can cause DNA damage and genomic instability (Shi and Jiang, 2021; Muto et al., 2023; Qin et al., 2023). Inflammatory mediators can suppress the function of immune cells such as T cells and NK cells (Vadakekolathu et al., 2020; Shi and Jiang, 2021; Muto et al., 2023; Qin et al., 2023). The main events impacted by p53 mutations or p53 loss are summarized in Figure 3.

### 2.3 TP53 and other hallmarks of AML

One of the hallmarks of cancer is uncontrolled cell proliferation; p53 controls cell division and prevents excessive proliferation (Mantovani et al., 2019). When DNA damage is detected, p53 halts the cell cycle to allow for repair, or it triggers apoptosis if the damage is too severe. Mutations in p53 render cells incapable of this checkpoint control, leading to uncontrolled proliferation (Mantovani et al., 2019; Capaci et al., 2020). In regard to the cancer hallmarks of stem-cell homeostasis and differentiation, p53 maintains a delicate balance between stem cell self-renewal and differentiation into mature cells (Shi and Jiang, 2021; Qin et al., 2023). It ensures the proper development of stem cells and prevents uncontrolled stem-cell expansion that could lead to tumors (Perez Montero et al., 2024; Scott et al., 2024).

Nutlin-3a, an MDM2 inhibitor and a selective activator of the p53 pathway, has been shown to exhibit dose-dependent antiproliferative and cytotoxic activity in OCI-AML-3 and MOLM-13 cells with wt p53 but to produce no response in HL-60 and NB4 cells expressing mutant p53 (Haaland et al., 2014; Borthakur et al., 2015; Trino et al., 2016; Fontana et al., 2021). The lack of response to Nutlin-3a indicated that the p53 pathway can be activated by Nutlin-3a only in cells with wt p53 (Kojima et al., 2005; Cheng et al., 2023; Zhou et al., 2023; Liu et al., 2024; Tomiyasu et al., 2024; Varineau and Calo, 2024).

In response to severe DNA damage or cellular stress, p53 triggers apoptosis, a process of controlled cell suicide. This eliminates potentially dangerous cells and prevents the spread of mutations (Cheng et al., 2023; Zhou et al., 2023; Liu et al., 2024; Tomiyasu et al., 2024; Varineau and Calo, 2024). Mutant p53 can malfunction in this pathway, allowing damaged cells to survive (Cheng et al., 2023; Zhou et al., 2023; Liu et al., 2024; Tomiyasu et al., 2024; Varineau and Calo, 2024). Furthermore, mutations that specifically keep the proliferation-promoting features or survival-preserving functions of wild-type p53, such as adaptation to metabolic stress, while disrupting the canonical tumor suppressive activities (such as apoptosis and senescence), can result in phenotypes that resemble those associated with gain-of-function mutations (Pfister and Prives, 2017). Controlling genome stability by p53 is another hallmark of AML and plays a central role in maintaining genomic integrity. It helps repair DNA damage through various mechanisms and activates checkpoints to halt cell division if DNA repair is incomplete (Vaddavalli and Schumacher, 2022). Dysfunction of p53 leads to an accumulation of mutations, increasing the risk of cancer (Wiederschain et al., 2005; Meyer et al., 2009), and alterations such as microsatellite instability or p53 mutations were enriched substantially in patients with therapy-related AML (Wiederschain et al., 2005; Meyer et al., 2009). Normal cells have a limited lifespan and eventually stop dividing after reaching a certain number of cell divisions (replicative senescence), but p53 can induce this senescence state in damaged or stressed cells, preventing them from becoming cancerous (Wiederschain et al., 2005; Meyer et al., 2009). Mutations in *TP53* can bypass this safeguard, allowing abnormal cell proliferation and leading to uncontrolled cell growth (Gutu et al., 2023). Research suggests a potential link between p53 function and lifespan regulation, indicating that heightened p53 activity is associated with a shorter lifespan as shown in murine models. Conversely, experiments in flies suggest that reduced p53 activity appears to extend lifespan (Wiederschain et al., 2005). *TP53* is well known as a “gatekeeper” of the cell cycle; it is capable of blocking cells in the G1/S phase if activated by hypoxia, heat shock, or other extrinsic or intrinsic stress signals (Olivier et al., 2010; Wang H. et al., 2023). In healthy cells, in case of DNA damage, ATM or ATR kinases activate checkpoint kinases (CHK1 and CHK2), which activate p53 and p21 and initiate G1 arrest and senescence phenotype (Wang H. et al., 2023). p53 activates cyclin-dependent kinase inhibitor 1A and p14 alternate open reading frame, which are involved in senescence and growth arrest, and targets several genes involved in the apoptosis and necrosis pathway (Barnoud et al., 2021). Mutations in *TP53* have shown partial or complete alteration of the target gene set, depending on the position and the type of mutation (Olivier et al., 2010; Barnoud et al., 2021; Kennedy and Lowe, 2022).

## 3 Current therapeutic strategies for *TP53* in AML

As previously mentioned, choosing and managing therapy for patients with AML who harbor *TP53* mutations or have complex karyotypes remains challenging (Abramowitz et al., 2017; Latif et al., 2021; Hassin and Oren, 2023). Moreover, the current therapeutic management of AML considers several risk factors that are included in prognostic models predicting therapy responses and outcomes, and these models classify patients with *TP53* mutations as poor responders (Haase et al., 2019). Here we will discuss the outcomes of patients with *TP53* mutations after treatment with selected therapy regimens (Olivier et al., 2010).

### 3.1 Intense chemotherapy

Intense chemotherapy is based on the combination of antimetabolic and antiproliferative agents like cytarabine and anthracyclines, and it is a backbone of AML therapy (DiNardo et al., 2019; Kadia et al., 2021). Intense chemotherapy based on high-dose cytarabine (AraC)/daunorubicin, known as the 7 + 3 regime, is still considered the gold standard of care (Kantarjian et al., 2021; Daver et al., 2022). Therapy modification by dose reduction of AraC or daunorubicine and the further introduction of fludarabine or cladribine has been shown to improve the response to therapy, patient outcomes, and therapy safety profile (Kadia et al., 2021). Intense chemotherapy is mainly followed by consolidation and/or maintenance therapy, and in some cases is followed by HSC transplantation (Guerra et al., 2019). A study has shown that drug-induced pancytopenia, which frequently occurs in patients treated with intense chemotherapy, became managable with protocol administration of granulocyte colony-stimulation factor (Kadia et al., 2021). Also, a new liposomal formulation of AraC/daunorubicin, known as CPX-351, has been shown to reduce the risk of cardiotoxicity in patients treated with that combination (Daver et al., 2020). However, none of these regimens specifically address the challenges of drug resistance linked to *TP53* (DiNardo et al., 2019; Daver et al., 2022). Mutations in the *TP53* gene render Acute Myeloid Leukemia (AML) resistant to traditional chemotherapies. Consequently, effective treatment strategies for *TP53*-mutated AML either bypass the need for wild-type p53 function altogether or aim to restore its normal activity (Daver et al., 2020). Most patients with *TP53*-mutated AML have a median overall survival of only a few months, despite receiving active anticancer treatment.

Counterintuitively, although chemotherapy and radiation (cytotoxic stress) aim to damage cancer cells, they do not directly cause *TP53* mutations (Yan et al., 2020). Individuals with *TP53* mutations in their blood stem cells (hematopoietic clones) face a significantly increased risk of developing Acute Myeloid Leukemia (AML). The median time to AML diagnosis after detecting a *TP53* mutation is approximately 4.9 years (Desai et al., 2019; Young et al., 2019; Saygin et al., 2023). While *TP53* mutations are rare in blood stem cells, these mutated cells have a survival advantage. This allows them to outcompete healthy cells under pressure from chemotherapy or stem cell transplant (Desai et al., 2019; Young et al., 2019; Saygin et al., 2023).

### 3.2 Hypomethylating agents

In the last decade, many hypomethylating agents (HMAs) have been developed for patients who cannot undergo intense chemotherapy, especially those over the age of 60 with high-risk features (Haase et al., 2019; Daver et al., 2023a; Wang H. et al., 2023). The most relevant approved drugs belonging to this category are azacytidine (AZA) and decitabine (DEC), which have gained considerable interest in the last decade due to the possibility of combining them with other types of therapies. (Daver et al., 2020; George et al., 2021; Kennedy and Lowe, 2022; Daver et al., 2023a; Wang H. et al., 2023). For instance, a 10-day decitabine regimen in patients with AML led to an excellent 100% ORR in patients with mutated *TP53* disease compared to 41% in those with wt *TP53* (*p* < 0.001), however was not sufficient for mutational clearance.

### 3.3 Allogenic stem cell transplantation

Allogenic stem cell transplantation (allo-SCT) is used in secondary AML following other therapies such as intense chemotherapy and HMA treatments. Allo-SCT can have a curative effect and can lower the probability of disease relapse in patients with a poor prognosis, but it requires early minimal residual disease (MRD) monitoring, and the incidence of complications is still high (Loke et al., 2022; Dekker et al., 2023; Gutman et al., 2023; Tettero et al., 2023). Genetic aberrations involving *TP53, FLT3, NPM1, RUNX1*, and *ASXL1*, together with factors such as age, sex, and cytogenetic characteristics, are the main risk factors affecting the outcome of patients undergoing allo-SCT (Yan et al., 2020; Loke et al., 2022; Malagola et al., 2023; Chattopadhyay et al., 2024; Park et al., 2024). *TP53* positive MRD status in patients with AML, for example, has been associated with a significantly worse survival (median overall survival, 6.4 months vs 21.7 months, *p* = 0.042) both in patients with *TP53*-mutated AML and myelodysplastic syndrome receiving HMA as frontline therapy (n = 24) prior allo-SCT (Olivier et al., 2010; Malagola et al., 2023; Song et al., 2023; Chattopadhyay et al., 2024; Park et al., 2024; Sahasrabudhe and Mims, 2024). Thus, the early detection of these mutations using complex MRD monitoring and testing for the loss of chimerism (LoC after allo-SCT is a serious concern as it can be an early indicator of relapse) after stem cell transplant are the key strategies for addressing the otherwise-poor survival of patients relapsing after allo-SCT (Sahasrabudhe and Mims, 2024).

### 3.4 Bcl-2 inhibitors

Bcl-2 family genes are composed of antiapoptotic and proapoptotic genes such as *Bcl-2* and *BAX/BAK*, respectively (DiNardo et al., 2019; Guerra et al., 2019). Bcl-2 inhibitor–based therapy relies on altering the equilibrium of Bcl-2 components by depleting the antiapoptotic members and allowing the p53-mediated activation of *BAX* and *BAK* following the permeabilization of the outer mitochondrial membrane and caspase cascade (DiNardo et al., 2019; Guerra et al., 2019; Carter et al., 2023a). Venetoclax (VEN) has proven to be as effective as monotherapy, especially in AML harboring *IDH1/2* and *SRSF2/ZRSF2* mutations (Daver et al., 2020; George et al., 2021; Thijssen et al., 2021). Further synergistic activity was observed with AZA in the VIALE-A trial and with low-dose cytarabine in the VIALE-C trial (Thijssen et al., 2021; Carter et al., 2023a; Malagola et al., 2023; Song et al., 2023; Pratz et al., 2024; Sahasrabudhe and Mims, 2024). However, the best outcome was obtained when VEN was combined with MCL-1 inhibitor in therapies (Thijssen et al., 2021; Casado and Cutillas, 2023). A cohort analysis of patients harboring *TP53* mutations demonstrated a lack of improvement among patients on the VEN + AZA regimen compared with historical controls (Hong et al., 2016; DiNardo et al., 2019). However, given the limited number of patients, further studies in this specific patient cohort are warranted. Although therapy with VEN + AZA has a good safety profile in elderly patients, the short response duration in patients with *TP53* mutation and rapidly acquired resistance to VEN warrants further investigations on the mechanisms of resistance in these patients as well as further work on novel combinatorial strategies (DiNardo et al., 2019; Guerra et al., 2019; Kadia et al., 2021; Zhang et al., 2022; Gutman et al., 2023). However, due to the rewiring of other BH3-mimetic family members upon Bcl-2 pharmacological inhibition, other therapeutic strategies with molecules targeting Bcl-2, Bcl-XL, and Bcl-W were evaluated. Navitoclax (ABT-263) is an antagonist of the antiapoptotic members of the Bcl-2 family—Bcl-2, Bcl-XL, and Bcl-W—and prevents their binding to the apoptotic effectors proteins Bax and Bak, thereby triggering apoptotic processes in cells overexpressing these proteins (Gress et al., 2024). However, the application of navitoclax in patients is challenging because its induction of severe thrombocytopenia limits its utility. Besides navitoclax, several other molecules have been tested in phase I and Ib clinical trials, including the Mcl-1 inhibitors AZD5991, MIK665, AMG176, and AMG397, alone or in combination with VEN (Luedtke et al., 2017; Tron et al., 2018; Carter et al., 2022; Fang et al., 2022; Liu et al., 2022; Carter et al., 2023a; Wang Y. et al., 2023; Torka et al., 2023). Furthermore, several dual inhibitors targeting Bcl-2/-XL have been developed, including LP-118, AZD0466, and dual Bcl-2/-XL PROTAC degraders (Khan et al., 2022; Gress et al., 2024). Finally, VEN has been tested with several FLT3 inhibitors such as quizartinib or gliteritinib in patients with FLT3-mutated AML as well as in those with wt FLT. VEN improved survival in a cohort of patients with mutated *FLT3*, successfully impairing leukemia progression (Yilmaz et al., 2022). While the list of potential combinations of BCL2 and FLT3 co-inhibition in acute myeloid leukemia tested preclinically expands rapidly, none of these so far has shown improved efficacy in patients with *TP53* mutations (Perl, 2019; Brinton et al., 2020; Carter et al., 2021; Zhu et al., 2021; Carter et al., 2023b; Popescu et al., 2023).

## 4 Future directions in the treatment of AML

In recent years, increased knowledge regarding the features about AML—obtained through genome-wide association studies, single-cell RNA sequencing, and proteomic approaches—has enabled the consideration of several novel therapeutic approaches for the treatment of patients with AML. These approaches could pave the way for new generations of HMA and BCL-2 inhibitors; such inhibitors are currently being evaluated as monotherapies or in combination with approved therapies (Daver et al., 2020).

### 4.1 *TP53* pathway interference

The main inhibitors of p53, MDM2 and MDM4, are often upregulated in many malignancies harboring wt p53 (Wang H. et al., 2023). While several molecules such as HDM201, MK-8242, BI-907828, RG7388, and RG7112 have been developed to interfere with p53 activity, most remain in the preclinical testing phase (Wang H. et al., 2023). Idasanutlin (RG7388), an MDM2 antagonist with a pyrrolidine structure, has demonstrated better efficacy, selectivity, and availability for the treatment of AML compared with the other drugs of the nutlin family (Trino et al., 2016; Fontana et al., 2021), and in a phase III trial with cytarabine, it improved survival and recovery in patients with R/R AML (Italiano et al., 2021; Konopleva et al., 2022; Daver et al., 2023b; Smalley et al., 2024). Mutant p53 tends to aggregate with p73, p63, or other p53 molecules, inactivating or reducing their activity (Kennedy and Lowe, 2022). Interference with these interactions would restore the activity of p73 and residual wtp53 (Ramos et al., 2020; Klein et al., 2021; Cai et al., 2022). ReACp53, for example, is a small peptide that interferes with the aggregation of mutant p53 with p73 and p63, and it has been shown to restore the wt conformation and nuclear localization of p53, promoting apoptosis *in vitro* and tumor suppression *in vivo* (Spaety et al., 2019; Zhang et al., 2019; Ramos et al., 2020; Klein et al., 2021; Rozenberg et al., 2021; Cai et al., 2022; Wang H. et al., 2023; Hassin and Oren, 2023).

### 4.2 *TP53* reactivation

Another strategy to address the severe outcomes of mutant *TP53* and the consequent LOF is to reverse the altered mutant conformation to one resembling that of wt *TP53* (Prokocimer et al., 2017; Hassin and Oren, 2023). Eprenetapopt (APR-246), also known as PRIMA1-MET, is a small molecule that causes selective apoptosis in cancer cells with *TP53* mutations (Hassin and Oren, 2023). Eprenetapopt binds covalently to cysteine residues in the DBD and forces conformational changes in the wt conformation, leading to the depletion of antioxidants and d-nucleotides and the induction of ROS(Reactive Oxygen Species)-linked cell death through ferroptosis (Sallman, 2020; Birsen et al., 2022; Daver et al., 2022). Eprenetapopt was evaluated with AZA in phase III clinical trial, but it showed no significant benefits in patients with *TP53* mutations (Daver et al., 2022; Hassin and Oren, 2023). In contrast, in combination with AZA and VEN, this combination therapy demonstrated an acceptable safety profile and promising signs of effectiveness. These findings support further investigation of this approach as a first-line treatment for *TP53*-mutated AML (Garcia-Manero et al., 2023; Wang H. et al., 2023). Other trial have evaluated APR-246 as post-transplant maintenance therapy and focused on patients with *TP53*-mutated acute myeloid leukemia (AML) or myelodysplastic syndromes (MDS) who had undergone allogeneic hematopoietic stem cell transplantation (allo-SCT) leading to encouraging RFS and OS outcomes in this high-risk population (Spaety et al., 2019). APR-246 was also evaluated with DEC, VEN, and low-dose cytarabine in patients with AML who were over 60 years old and ineligible for intense chemotherapy. The drug combination produced encouraging results and had an acceptable safety profile (Mishra et al., 2022).

In addition, APR-548, an orally available *TP53* reactivator, has undergone clinical evaluation in patients with solid tumors and hematological malignancies, indicating that p53 mutants differ in functionality and form from typical AML cases and subsequently display inconsistent responses to therapy with APR-548 at the cellular level (George et al., 2021).

Finally, ZMC1, ZMC2, and ZMC3, which belong to a new class of zinc metallochaperones, sequester zinc ions crucial for DNA recognition from mutated p53, promoting wt-like behavior, p53-dependent apoptosis *in vitro*, and tumor regression *in vivo* (Wang H. et al., 2023; Hassin and Oren, 2023). These zinc metallochaperones have been shown to reactivate mutant p53 using an on/off switch, and they have shown specificity for mutant p53 (Hassin and Oren, 2023).

### 4.3 Trisenox

Trisenox (ATO) (As_2_O_3_) is a small molecule that binds to allosteric sites on a wide subset of p53 mutants and induces p53 proteasome–mediated degradation via structural stabilization (Gummlich, 2021; Song et al., 2023). It has been observed that a few p53 mutants treated with ATO demonstrate restored wt p53 activity (Daver et al., 2022; Hassin and Oren, 2023). Trisenox is mainly used in certain subtypes of AML, including acute promyeloid leukemia, in which it has shown dose-dependent dual effects, including differentiation at low concentrations and apoptosis at high concentrations, on Leukemia Stem Cells (LSC) (Yilmaz et al., 2021). Clinical trials involving treatments with trisenox + HMA + all-trans retinoic acid are ongoing (Yilmaz et al., 2021).

### 4.4 Chimeric antigen receptor T cells

Development of adoptive T cell therapy for relapsed/refractory acute myeloid leukemia (R/R AML) has shown limited progress to date. Chimeric antigen receptors (CARs) implemented *in vitro* in T cells have proven to be effective for R/R B-cell lymphoid malignancies (CD19^+^) and multiple myeloma (Caruso et al., 2022; Gurney and O’Dwyer, 2021; Mueller et al., 2024). Several proteins commonly overexpressed in AML, such as CD38, CD123, TIM3, CD7, CD19, and NKG2D, have been considered as targets for evaluation in AML treatments, with the aim of treatment being to eradicate the LSC-like population (Gurney and O’Dwyer, 2021; Valeri et al., 2022; Cao et al., 2022; Cui et al., 2021; El et al., 2021; Jetani et al., 2021; Mai et al., 2023). However, it has been difficult to find suitable tumor-associated antigens for CAR T-cell administration in patients with AML (Cui et al., 2021; El et al., 2021; Yilmaz et al., 2021; Sanford et al., 2023); thus, clinical responses to CAR T-cell therapy are seen in only one-fourth of treated subjects (Atilla and Benabdellah, 2023). Recent studies have tried to overcome *TP53* deficiency–linked resistance to CAR T cells by targeting the lipids metabolism aiming at blocking cholesterol metabolism or activity of carnitine o-octanoyltransferase and at improving CAR T-cell anti-leukemic properties. While the specific results of targeting these pathways are not provided here, further investigation might lead to more effective and personalized treatment options in the future. (Mueller et al., 2023; Roche et al., 2023; Sanford et al., 2023; Albinger et al., 2024). Although CAR T-cell therapy alone might not be sufficient to achieve a complete eradication of residual disease, some studies suggest that combined CAR T-cell therapy and pharmacological blockade with demethylating agents or venetoclax (VEN) might be a promising strategy. This approach could lead to more effective and better-tolerated cellular therapies for patients with *TP53*-mutated myeloid neoplasms (Leick et al., 2022; Mandeville et al., 2023; Mueller et al., 2023; Sanford et al., 2023).

### 4.5 CAR NK cells

NK cells are the first line of defense against tumor cells (Mandeville et al., 2023; Albinger et al., 2024). They induce cell death in two ways: by releasing tumor necrosis factor-α and IFN-γ, which activate the extrinsic apoptosis pathway, and by triggering cell death using the tumor necrosis factor–related apoptosis-inducing ligand or Fas ligand. Compared to the use of CAR T cells, the use of CAR NK cells has significantly fewer side effects (Gurney and O’Dwyer, 2021; Albinger et al., 2024; Albinger et al., 2022). Until now, CAR NK therapy has shown good efficacy in clinical trials against circulating AML cells, but it displays a low penetrative ability in bone marrow niches; thus, it is considered as a maintenance or consolidation therapy before or following an allo-SCT rather than as induction therapy (Gurney and O’Dwyer, 2021). After intensive therapy and allogenic hematopoietic cell transplantation, the outcomes of CAR NK cell therapy in AML patients with *TP53* mutations remains poor, those patients with lower *TP35* VAFs at diagnosis might still benefit from transplantation. combined with CAR NK therapy (Zhao et al., 2023). One possible mechanism of overcoming resistance to CAR NK therapy would be to select AML clones that resist or even suppress NK cell activity and mobilize them from Bone marrow, making them more susceptible to therapy (Gurney and O’Dwyer, 2021; Valeri et al., 2022; Albinger et al., 2024; Li et al., 2023). Persistent hypoxia and bone marrow remodeling with poor vascularization might also be obstacles to the efficacy of CAR NK therapy (Caruso et al., 2022). These and other factors contributing to therapy resistance need further investigation to improve the chances of disease eradication.

### 4.6 Immunotherapy

Since the approval of gemtuzumab ozogamicin (GO, a monoclonal antibody targeting CD33, conjugated with calicheamicin) (Laszlo et al., 2019; Bouvier et al., 2021; Casado and Cutillas, 2023), immunotherapy for AML has advanced substantially. New monoclonal antibodies, used along with other types of therapies in the induction phase or as a part of consolidation therapy after intense chemotherapy, have been added to the available treatment options (Hassin and Oren, 2023). For example, sabatolimab (MBC453) is a humanized, high-affinity, IgG4, anti-TIM3 antibody that uses an autocrine signaling loop via galactin-9 and promotes LSC renewal; it is currently being combined with HMA in an ongoing clinical trial (Brunner et al., 2024; Zeidan et al., 2024). Its observed side effects have been minimal, and the parameters of recovery and patients’ OS have been encouraging, especially in patients with AML harboring mutations such as *RUNX1* and *ASXL1* (Kantarjian et al., 2021; Daver et al., 2022). Another antibody, magrolimab, is a humanized antibody against CD47, a surface receptor expressed by myeloid malignancies that helps tumor cells evade phagocytosis (Bouvier et al., 2021; Zhao et al., 2023). Magrolimab has been evaluated in AML patients not eligible for intense chemotherapy who were in the early stages of treatments with AZA + VEN, and it demonstrated good tolerability, but it also caused frequent side effects such as anemia and fatigue (George et al., 2021; Zeidan et al., 2022; Daver et al., 2023c).

Bispecific and trispecific antibodies are a promising new area of immunotherapy for AML (Arvindam et al., 2021; Bouvier et al., 2021; Boyiadzis et al., 2023). These engineered molecules, which offer a targeted approach of attacking AML cells by harnessing the immune system, include flotetuzumab (targets CD33 on AML cells and CD16 on NK cells), AMG 330 (targets CD33 on AML cells and CD3 on T cells), and JNJ-63709178 (targets CD33; bispecific antibody) (Krupka et al., 2016; Jitschin et al., 2018; Laszlo et al., 2019; Vadakekolathu et al., 2020; Uy et al., 2021; Barwe et al., 2022; Boyiadzis et al., 2023; Marcinek et al., 2023; Rimando et al., 2023). Study investigating the CD123 × CD3 bispecific, dual-affinity, retargeting antibody flotetuzumab (CP-MGD006-01; NCT02152956) demonstrated complete remission in almost 50% of patients with *TP53*-mutated, R/R AML, and these patients had significantly higher tumor inflammation signature, FOXP3, CD8, inflammatory chemokine, and PD1 gene expression scores at baseline compared with nonresponders (Vadakekolathu et al., 2020). Examples of relevant clinical trials are given in Table 2.

**TABLE 2:** Examples of clinical trials in acute myeloid leukemia with the potential to target *TP53*.

Trial code	Phase	Approach type	Evaluated therapy
NCT05455294	Phase I	HMA + Bcl-2 inhibitors	DEC + VEN + navitoclax
NCT03745716	Phase III	HMA + mutTP53 alteration	AZA + APR-246
NCT03855371	Phase I	HMA + small molecule	DEC + ATO
NCT04638309	Phase I	HMA + mut*TP53* alteration	AZA + APR-548
NCT05297123	Phase I	Small-molecule combination	ATO + ATRA
NCT03766126	Phase I	Cell-mediated	CD123 CAR-T
NCT04678336	Phase I	Cell-mediated	CD123 CAR-T
NCT02944162	Phase I-II	Cell-mediated	CD33 CAR-NK cells
NCT04623944	Phase I	Cell-mediated	NKX101-CAR NK
NCT01217203	Phase I	Antibody	IPH2101 + lenalidomide (anti-KIR2D+)
NCT01687387	Phase II	Antibody	Lirilumab (anti-KIR2D)
NCT01714739	Phase I-II	Antibodies (PD-1, KIR2, CTLA-4)	Nivolumab + Lirilumab/ipilimumab
NCT02848248	Phase I	Antibody-drug conjugate	SGN-CD123A
NCT05396859	Recruiting	Cytidine deaminase inhibitors	Entrectinib + ASTX727
NCT02545283	Terminated	MDM2 antagonist	Idasanutlin + cytarabine

HMA, Hypomethylating agent; DEC, Decitabine (a specific HMA, drug); ATO, Arsenic trioxide (a small molecule drug); AZA, Azacitidine (another HMA, drug); APR-548, A small molecule drug targeting specific pathways; ATRA, All-trans retinoic acid (a small molecule drug); CD123 CAR-T, Chimeric antigen receptor T cells engineered to target the CD123 protein on leukemia cells; CD33 CAR-NK, cells, Chimeric antigen receptor Natural Killer cells engineered to target the CD33 protein on leukemia cells; NKX101- CAR NK, Specific brand name for CAR-NK, cells targeting a certain molecule; IPH2101, A specific antibody targeting the KIR2D + receptor; Lenalidomide, An immunomodulatory drug; Lirilumab, antibody targeting the KIR2D + receptor; Nivolumab, antibody targeting the PD-1, checkpoint protein; Lirilumab/Ipilimumab, Antibodies targeting KIR2D+ and CTLA-4, checkpoint protein, respectively; Antibody-drug conjugate (ADC): an antibody linked to a chemotherapy drug that delivers the drug directly to cancer cells; SGN-CD123A, A specific type of ADC, targeting the CD123 protein; Cytidine deaminase inhibitors, Drugs that block the enzyme cytidine deaminase; Entrectinib, A small molecule drug targeting specific mutations; ASTX727, Another small molecule drug targeting specific pathways; MDM2 antagonist, Drug that blocks the interaction between MDM2 and p53 protein; Idasanutlin, specific MDM2 antagonist; Cytarabine, chemotherapy drug.

## 5 Conclusion

For over four decades, high-dose cytotoxic chemotherapy remained the mainstay treatment for AML. However, recent scientific breakthroughs have revolutionized our understanding of this leukemia’s molecular basis. This newfound knowledge has not only shed light on the underlying causes of AML but also led to the development of several targeted therapies. These novel agents offer greater efficacy and reduced toxicity compared to conventional chemotherapy. With novel compounds selectively targeting *TP53*, therapeutic approaches targeting the *TP53* pathway are progressing in early clinical testing and could soon be considered the standard of care for individuals with AML who are 60 and older and ineligible for intense therapy or who have been diagnosed with advanced disease. Moreover, many targeted approaches and combinations are currently being tested in clinical trials with the aims of reducing the rate of disease recurrence and minimizing drug toxicities, making the prospects of new AML therapies promising. However, further investigation of the factors contributing to therapy resistance is warranted, and efforts to understand the metabolic and immune mechanisms contributing to therapy failure with the help of single-cell, high-throughput technology and spatial analysis are ongoing.

## 6 Simple summary

Acute myeloid leukemia (AML), an aggressive malignancy of hematopoietic stem cells, is associated with poor outcomes, especially in elderly patients, due to several genetic and chromosomal aberrations. Tumor protein p53 (*TP53*) is a key tumor-suppressor gene involved in a variety of cellular processes, including the regulation of apoptosis, metabolism, and the rewiring of the immune environment. Although *TP53* mutations are relatively rare in patients with *de novo* AML, these mutations has been identified as an important molecular subgroup, and patients with these mutations have the worst prognosis and shortest overall survival among patients with AML, even when treated with aggressive chemotherapy and allogeneic stem cell transplant for relapsed or therapy-related AML. Progress in AML genetics and biology has brought the novel therapies, however, the clinical benefit of these agents for patients whose disease is driven by *TP53* mutations remains largely unexplored. This review focuses on examining the role of *TP53* mutations on such hallmarks of leukemia like metabolic rewiring and immune evasion, the clinical significance of these changes, and the current progress in the therapeutic targeting of mutated p53 and its downstream effects.
